# Shorter duration of first-line chemotherapy reflects poorer outcomes in patients with HER2-negative advanced breast cancer: a multicenter retrospective study

**DOI:** 10.1038/s41598-021-00711-x

**Published:** 2021-11-02

**Authors:** Shogo Nakamoto, Junichiro Watanabe, Shoichiro Ohtani, Satoshi Morita, Masahiko Ikeda

**Affiliations:** 1 Division of Breast Oncology, Shizuoka Cancer Center, 1007 Shimonagakubo, Nagaizumi, Shizuoka 411-8777 Japan; 2 Division of Breast and Thyroid Gland Surgery, Fukuyama City Hospital, 5-23-1 Zao, Fukuyama, Hiroshima 721-8511 Japan; 3 Department of Breast Oncology, Juntendo University School of Medicine, 3‑1‑3 Hongo, Bunkyo‑ku, Tokyo, 113‑8431 Japan; 4 Division of Breast Surgery, Hiroshima City Hiroshima Citizens Hospital, 7-33 Motomachi, Naka-ku, Hiroshima, Hiroshima 730-8518 Japan; 5 Division of Biomedical Statistics and Bioinformatics, Kyoto University Graduate School of Medicine, Yoshida-Konoe, Sakyo-ku, Kyoto, 606-8501 Japan

**Keywords:** Breast cancer, Cancer therapy, Metastasis

## Abstract

Post-progression survival affects overall survival (OS) in patients with HER2-negative advanced breast cancer (HER2-ABC); thus, the optimal choice of first-line chemotherapy (1LCT) remains controversial. We investigated patients with HER2-ABC focusing on their sensitivity to 1LCT. We retrospectively analyzed patients with HER2-ABC who received 1LCT between January 2011 and December 2016 in three participating institutions. We identified 149 patients in the shorter and 152 patients in the longer time to treatment failure (TTF) groups. The median OS was significantly longer in the longer TTF group (hazard ratio [HR] 0.44, *P* < 0.001, log-rank). In the shorter TTF group, OS of patients who received paclitaxel plus bevacizumab (PB) therapy was significantly inferior to that of those who received chemotherapy other than PB (HR 2.57, *P* < 0.001, log-rank), and subsequent eribulin therapy significantly improved OS from 1LCT initiation (Wilcoxon *P* < 0.001); multivariate analyses showed that 1LCT PB therapy was an independent risk factor for poorer OS (HR 2.05, *P* = 0.003), while subsequent eribulin therapy was an independent prognostic factor for better OS (HR 0.56, *P* = 0.004). OS was significantly poorer in patients with HER2-ABC with a shorter duration of 1LCT, including PB therapy, while subsequent eribulin therapy improved OS.

## Introduction

Over the past decade, many new anticancer agents have been developed and at present, there are a variety of treatment options for managing HER2-negative advanced breast cancer (HER2-ABC). Although the new agents have improved progression-free survival (PFS), only a few agents and regimens have demonstrated an improvement in overall survival (OS) in the first-line setting. This difference is because OS can be affected by the response to subsequent therapies, increased tumor load, and relatively longer post-progression survival^1,2^, and several reports have indicated that PFS is not a good surrogate for OS^3,4^.

Recently, it has been reported that first-line treatment with certain new agents prolonged not only PFS but also OS. The CLEOPATRA study showed that adding pertuzumab to trastuzumab and docetaxel, compared with the addition of placebo, significantly improved PFS and OS^5^. Palbociclib, an inhibitor of cyclin-dependent kinases 4 and 6, in combination with letrozole as first-line treatment led to a significant improvement in PFS and OS compared with patients treated with letrozole alone in a real-world population^6^. Unlike patients with HER2-positive ABC or estrogen receptor (ER) -positive HER2- ABC in earlier phases of illness, there is currently no consensus on the choice of first-line chemotherapy (1LCT) for HER2-ABC in the guidelines^1,7^. Thus, the 1LCT regimen is chosen according to the patient’s performance status, age, comorbidity, tumor load, previous therapy, and preferences^1,7^.

The Epidemiological Strategy and Medical Economics database demonstrated significant improvements in PFS and OS with paclitaxel + bevacizumab (PB) as first-line chemotherapy for HER2-ABC in a real-world setting^8^. The results indicated that a long-term response to first-line treatment might be associated with improved OS. Furthermore, several studies have reported a positive association between the duration of first-line chemotherapy and the length of survival^9,10^. A systematic review of randomized controlled trials showed that a longer duration of first-line chemotherapy is associated with longer OS^9^. On the other hand, a retrospective study using real-world data showed that a shorter first-line treatment duration (less than 6 months) was significantly related to the unfavorable survival of patients with ABC^10^. Thus, the short-term response to first-line treatment might be associated with a poorer OS.

Our previous study^11^ showed that PB therapy as 1LCT improved the overall response rate (ORR) and time to treatment failure (TTF) compared with conventional chemotherapy in patients with HER2- ABC after propensity score matching. However, despite improvements in OS in several patient subgroups, no OS benefit with PB therapy was seen in the overall patient population^11^. Therefore, we hypothesized that patients who did not respond to PB therapy might have poorer OS than those who did not respond to conventional chemotherapy, resulting in no overall OS benefit. Thus, we focused on the clinical course of patients with a short-term response to first-line treatment.

In this multicenter retrospective study, we investigated HER2-ABC patients with a focus on sensitivity to 1LCT. We evaluated the association between the duration of 1LCT and OS and the clinical course of patients with a short duration of 1LCT in HER2- ABC. Furthermore, we investigated the factors leading to better OS in patients with a short duration of 1LCT.

## Results

### Patient characteristics

The study included 301 HER2-negative ABC patients at three institutions, with 149 patients in the shorter TTF group and 152 patients in the longer TTF group. Of the total patients, 114 were in the PB group and 187 were in the non-PB group. ER-positive patients numbered 77 (67.5%) in the PB group and 134 (71.7%) in the non-PB group. The regimens in the non-PB group were anthracycline in 24 cases, taxane in 28, eribulin in 23, 5-FU in 99, and other regimens in 13^11^. The characteristics at baseline (at the initiation of 1LCT) are shown in Table 1. Among patients with ER-positive ABC, 51 patients (51/99: 51.5%) in the shorter TTF group and 68 patients (68/112: 60.7%) in the longer TTF group received endocrine therapy for advanced breast cancer treatment before 1LCT. Compared with the longer TTF group, patients in the shorter TTF group included more ER-negative patients (31.5% vs. 20.4%, *P* = 0.048), patients with a shorter disease-free interval (DFI), i.e., < 24 months (67.1% vs. 48.7%, *P* = 0.002), and fewer patients who had received PB therapy (30.9% vs. 44.7%, *P* = 0.017). More detailed characteristics of the patients at the baseline of first-line chemotherapy are shown in Supplementary Table S1.

**Table 1:** Patient characteristics at baseline.

	short TTF, *n*	%	long TTF, *n*	%	*P* value
Total	149		152		
**Median age, years (range)**	58 (28–87)		60 (29–90)		0.045^a^
≥ 60 years	69	46.3	77	50.7	0.49
**Estrogen receptor status**					
Positive	99	66.4	112	73.7	0.048^b^
Negative	47	31.5	31	20.4	
Unknown	3	2.0	9	5.9	
**Diagnosis**					
De novo	51	34.2	44	28.9	0.39
Recurrence	98	65.8	108	71.1	
**Metastases**					
Central nervous system	8	5.4	9	5.9	1.00
Bone	89	59.7	87	57.2	0.73
Lung	51	34.2	61	40.1	0.34
Pleura/ lymphangiopathy	33	22.1	34	22.4	1.00
Lymph node	105	70.5	101	66.4	0.46
Liver	53	35.6	57	37.5	0.81
**Type of metastases**					
Visceral	95	63.8	77	59.7	0.81
Non-visceral	54	36.2	53	34.9	
**Number of metastatic sites**					
≥ 3	91	61.1	84	55.3	0.35
< 3	58	38.9	68	44.7	
**Perioperative chemotherapies **^**c**^					
Yes	75	50.3	72	47.4	0.65
No	74	49.7	80	52.6	
**Disease-free interval**					
< 24 months	100	67.1	74	48.7	0.002
≥ 24 months	49	32.9	78	51.3	
**Prior endocrine therapy **^d^	51	51.5	68	60.7	0.077
**First-line chemotherapy**					
PB therapy	46	30.9	68	44.7	0.017
Other chemotherapies	103	69.1	84	55.3	
**Response of first-line therapy**					
Overall response rate	49	32.9	101	66.4	< 0.001
Clinical benefit rate	51	34.2	148	97.1	< 0.001

*PB* paclitaxel plus bevacizumab, *TTF* time to treatment failure.

^a^Wilcoxon’s rank-sum test was performed.

^b^Comparing estrogen receptor positive and estrogen receptor negative.

^c^Treatment included anthracycline and/or taxane.

^d^Endocrine therapy for advanced breast cancer treatment before first-line chemotherapy in patients with estrogen receptor positive.

### OS

**Figure 1:**
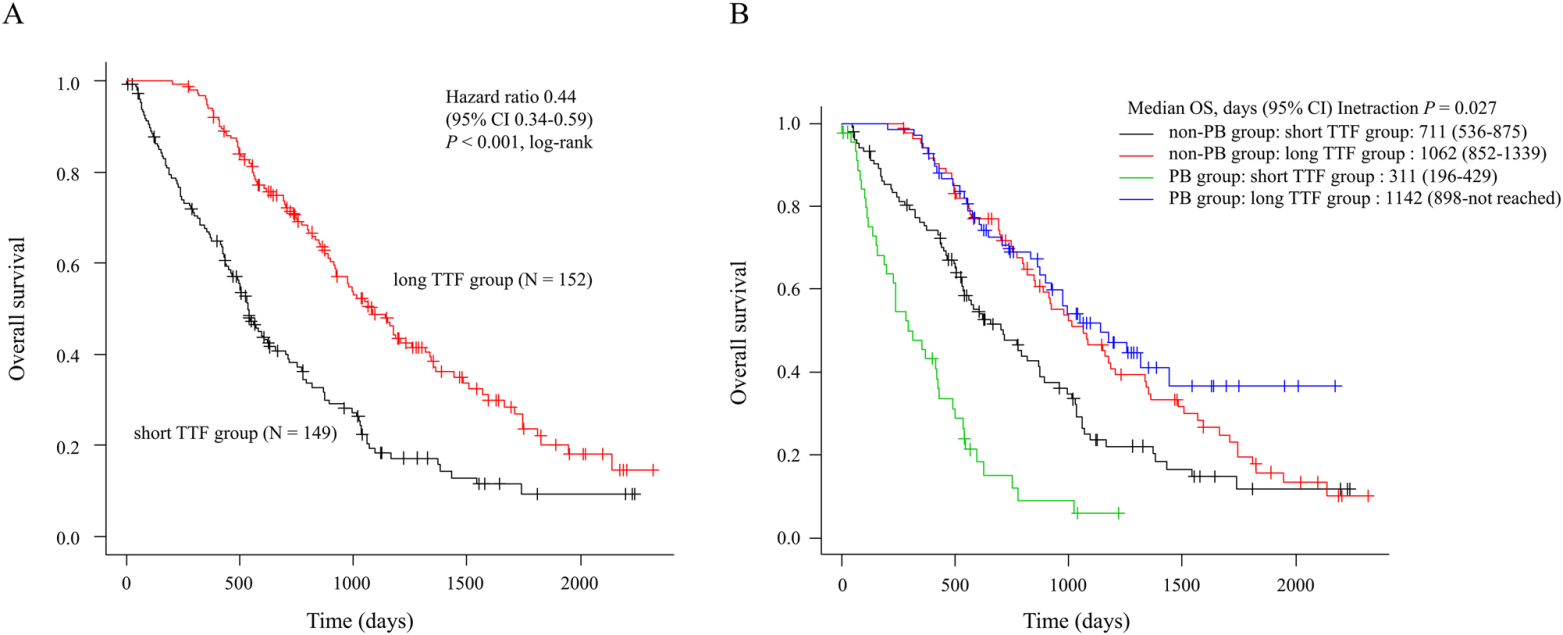
Overall survival according to the duration of first-line time to treatment failure (**A**) and overall survival based on the duration of first-line time to treatment failure and first-line chemotherapy regimens (**B**). *CI*, confidence interval, *HR* hazard ratio, *PB* paclitaxel plus bevacizumab, *TTF* time to treatment failure.

The median OS from the initiation of 1LCT was significantly longer in the longer TTF group than in the shorter TTF group (1080 vs. 536 days; HR 0.44; 95% confidence interval [CI] 0.34–0.59, log-rank *P* < 0.001: Fig. 1A). OS was significantly poorer in the PB group than in the non-PB group in patients with a shorter TTF (311 vs. 711 days; HR 2.57; 95% CI 1.71–3.85, log-rank *P* < 0.001) (Fig. 1B). In contrast, there was no difference in OS between PB and non-PB groups with longer TTF (1142 vs. 1062 days; HR 0.80, 95% CI 0.52–1.22; *P* = 0.29, log-rank) (Fig. 1B).

### Factors associated with a longer TTF of first-line chemotherapy.

We evaluated the factors for longer TTF of 1LCT. Logistic regression analysis showed that shorter DFI (< 24 months) significantly increased the risk of a shorter TTF (*P* = 0.011, Table 2). When compared by first-line chemotherapy regimen, logistic regression analyses showed that patients with shorter DFI (< 24 months) in the PB group and patients with ≥ 3 metastatic sites at the initiation of 1LCT in the non-PB group were at increased risk of a shorter TTF (*P* = 0.024 and *P* = 0.023, respectively, Supplementary Table S2).

**Table 2:** The relative risk of longer time to treatment failure of first-line chemotherapy according to patient and tumor characteristics (logistic regression).

Characteristics	OR	95% CI	P value
Age at first-line chemotherapy	1.02	0.99–1.04	0.15
Estrogen receptor negative	0.92	0.49–1.73	0.80
Disease-free interval < 24 months	0.49	0.28–0.85	0.011
Prior endocrine therapy ^a^	1.82	0.98–3.39	0.059
First-line PB therapy	1.37	0.75–2.49	0.30
ORR of first-line therapy	4.94	2.76–8.83	< 0.001

*CI* confidence interval, *OR* Odds ratio, *ORR* overall response rate, *PB* paclitaxel plus bevacizumab.

^a^Endocrine therapy for advanced breast cancer treatment before first-line chemotherapy.

### Association between TTF and OS

As a whole, a moderate, but significant positive correlation between TTF and OS (ρ ↑　(Spearman's ρ ) = 0.53, *P* < 0.001) was observed in this study. We conducted further analyses regarding the correlation according to 1LCT regimens. The PB group tended to have a stronger correlation than the non-PB group (ρ ↑　(Spearman's ρ ) = 0.67, *P* < 0.001 and ρ  ↑　(Spearman's ρ )= 0.47, *P* < 0.001, respectively).

### Impact of eribulin and paclitaxel PB therapy on OS in the shorter TTF group

**Figure 2:**
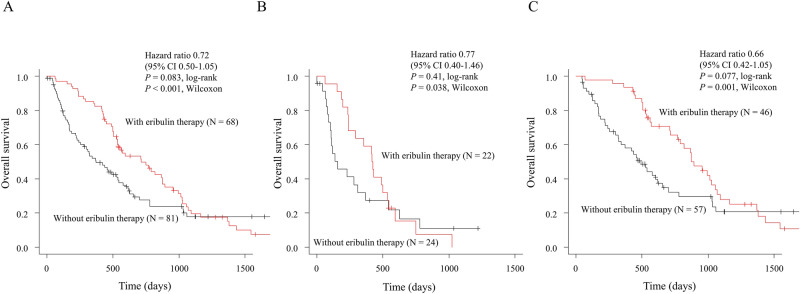
Overall survival with and without eribulin therapy in the shorter time to treatment failure group (**A**), in the PB group (**B**), and in the non-PB group (**C**). *CI* confidence interval, *HR* hazard ratio, *PB* paclitaxel plus bevacizumab.

We performed further analyses to determine the prognostic factors for 1LCT and we additionally evaluated the utility of eribulin as subsequent treatment (i.e., 2nd or ≥ 3rd line chemotherapy). In the shorter TTF group, eribulin therapy significantly improved OS (716 vs. 371 days; *P* < 0.001, Wilcoxon, Fig. 2A). And in the same group, a significant improvement in OS was observed in both the PB (421 vs. 155 days; *P* = 0.004, Wilcoxon, Fig. 2B) and the non-PB subgroups; (875 vs. 493 days; *P* = 0.001, Wilcoxon, Fig. 2C). We performed univariate and multivariate analyses to evaluate independent risk factors for OS (Table 3). In the multivariate analyses, first-line PB therapy was an independent risk factor for poorer OS (HR, 2.05; 95% CI, 1.27–3.29; *P* = 0.003), and eribulin, as subsequent treatment, was an independent factor for better OS (HR, 0.56; 95% CI, 0.37–0.83; *P* = 0.004), regardless of ER (negative vs. positive) and endocrine therapy after 1LCT (yes vs. no).

**Table 3:** Univariate and multivariate analyses of the overall survival in patients with the shorter time to treatment failure group (Cox hazard model).

Variables	Univariate			Multivariate		
	HR	95% CI	*P*	HR	95% CI	*P*
Age (≥ 60 vs. < 60 years)	1.04	0.72–1.51	0.82	–	–	–
Estrogen receptor (negative vs. positive)	1.75	1.19–2.59	0.005	1.17	0.70–1.96	0.54
Diagnosis (recurrence vs. advanced)	1.46	0.97–2.18	0.067	1.18	0.51–2.72	0.70
**Metastatic sites (yes vs. no)**						
Central nervous system	5.85	2.43–14.1	< 0.001	3.41	1.31–8.85	0.012
Bone	1.47	1.00–2.15	0.050	1.43	0.93–2.21	0.10
Lungs	1.15	0.78–1.69	0.48	–	–	–
Pleura and/or lymphangiopathy	1.55	1.00–2.39	0.049	2.11	1.24–3.61	0.006
Lymph nodes	1.19	0.79–1.77	0.41	–	–	–
Liver	2.06	1.41–3.01	< 0.001	2.56	1.53–4.27	< 0.001
Visceral metastasis (yes vs. no)	1.95	1.30–2.91	0.001	0.91	0.51–1.64	0.76
Number of metastatic sites (≥ 3 vs. < 3)	1.57	1.06–2.31	0.024	1.05	0.60–1.85	0.86
Perioperative chemotherapy ^a^ (yes vs. no)	1.91	1.31–2.79	< 0.001	2.38	1.17–4.83	0.017
Disease-free interval (< 24 months vs. ≥ 24)	1.43	0.95–2.15	0.085	2.03	1.17–3.50	0.011
First-line chemotherapy regimens (PB vs. non-PB)	2.57	1.71–3.85	< 0.001	2.05	1.27–3.29	0.003
Eribulin as subsequent therapy (yes vs. no)	0.72	0.50–1.05	0.085	0.56	0.37–0.83	0.004
Endocrine therapy after first-line chemotherapy (yes vs. no)	0.43	0.28–0.65	< 0.001	0.40	0.23–0.71	0.002

*CI* confidence interval, *HR* hazard ratio, *PB* paclitaxel plus bevacizumab.

### Impact of eribulin and paclitaxel PB therapy on OS in the longer TTF group

**Figure 3:**
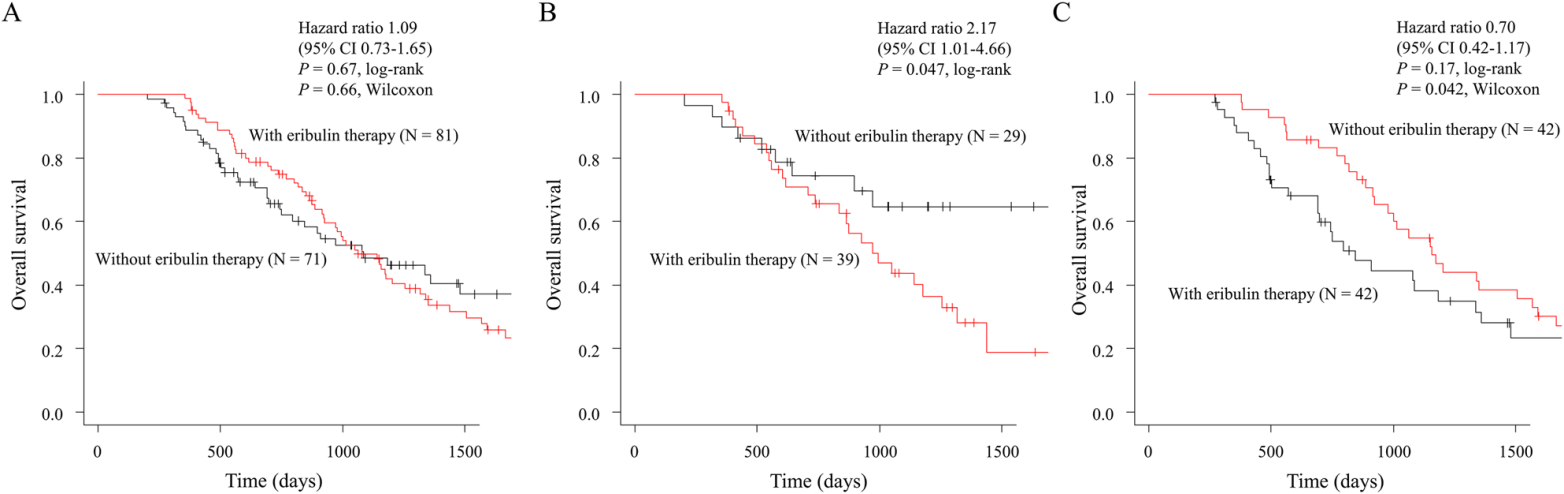
Overall survival with or without eribulin therapy in the overall longer time to treatment failure group (**A**), in the PB group (**B**), and in the non-PB group (**C**). *CI* confidence interval, *HR* hazard ratio, *PB* paclitaxel plus bevacizumab.

In the longer TTF group, eribulin therapy did not improve OS (1062 vs. 1085 days; HR, 1.09; 95% CI, 0.73–1.65; *P* = 0.67, log-rank: Fig. 3A). However, the results differed between the PB and the non-PB subgroups. In the PB subgroup, eribulin therapy did not improve OS (995 days vs. not reached; *P* = 0.042, log-rank: Fig. 3B), while OS was improved in the non-PB subgroup (1158 vs. 846 days, *P* = 0.042, Wilcoxon: Fig. 3C). The breakdown of univariable and multivariable analyses to identify risk factors for poorer OS is shown in Supplementary Table S3. Unlike the shorter TTF group, first-line PB therapy and eribulin as subsequent treatment were not associated with OS.

## Discussion

In this study, a moderately significant correlation was observed between TTF and OS, and OS was significantly poorer in the shorter TTF group than in the longer TTF group. In the shorter TTF group, first-line PB therapy was associated with the significantly inferior OS than conventional chemotherapy, and subsequent eribulin was significantly associated with better OS. Our results suggest that eribulin improves OS in HER2- ABC patients with resistance to 1LCT including PB therapy.

Although OS is the primary endpoint to assess the efficacy of new treatments in solid tumors including ABC, the analysis of OS requires larger sample sizes and prolonged follow-up of all patients. Thus, the evaluation of new treatments might be delayed^1–4^. In addition, the potential effects of first-line therapies on OS can be attenuated by the effects of subsequent therapy^1,3,12^. For example, in metastatic colorectal cancer, improved PFS of first-line chemotherapy is strongly associated with improved OS^13^, and PFS is an acceptable surrogate for OS^13,14^. However, in ABC, a meta-analysis showed that time to progression might be a useful surrogate for predicting survival in patients receiving first-line anthracycline-based chemotherapy^15^. A broad-based structured review and meta-analysis of randomized trials demonstrated a significant association between disease progression and survival in patients with ABC and suggested that treatment that prolongs time to progression can lead to longer OS than an alternate treatment^12^. Our results also demonstrated a moderate but positive correlation between TTF and OS and were identical to previous studies^12,15^. Thus, physicians must choose the appropriate first-line chemotherapy to achieve a longer TTF. In contrast, several meta-analyses concluded that PFS was not an acceptable surrogate endpoint in the first-line treatment setting^3,4^. Therefore, it has been controversial whether PFS can be considered a good surrogate for OS to assess the efficacy of first-line treatment for patients with ABC^3,4,12,15^.

On the other hand, a retrospective study showed that a shorter duration (less than 6 months) of first-line treatment was significantly associated with unfavorable survival^10^. We showed that a shorter TTF (less than 183 days) was significantly associated with poorer OS than that of patients with a longer TTF, suggesting that the short-term response to first-line treatment might be associated with poorer OS. Tumor chemoresistance is associated with low efficacy of chemotherapy^16,17^ and might be one of the main causes of short-term response to first-line treatment in this study. For example, a multidrug resistance system that surviving tumor cells acquire after chemotherapy treatment promotes the efflux of anticancer drugs from tumor cells, reducing drug absorption^17^. In addition, tumor chemoresistance can be associated with various mechanisms including interactions among cancer cells and the tumor microenvironment^16,17^. The mechanisms involve cancer cell heterogeneity, cancer stem cells, cancer-associated macrophages, immune cell modulation, hypoxia, and epithelial-mesenchymal transition (EMT), and can modify the tumor microenvironment during chemotherapy, leading to chemoresistance^17^.

Bevacizumab is a humanized monoclonal antibody directed against vascular endothelial growth factor (VEGF)^18^. VEGF inhibition with monoclonal antibodies such as bevacizumab slows tumor growth by limiting the formation of new blood vessels in tumor cells and therefore limiting their blood supply^19^. Several clinical trials have demonstrated improvements in ORR and PFS compared with a control arm^8,18,20,21^. However, it has been reported that bevacizumab leads to poorer outcomes^20,22–25^. In our study, in the shorter TTF group, patients receiving PB therapy had significantly poorer OS than those who received non-PB therapy (P < 0.001, log-rank), and first-line PB therapy was an independent risk factor for poorer OS (HR, 2.05; *P* = 0.003). In contrast, there were no differences in the longer TTF group (log-rank *P* = 0.29). In the E2100 study, the authors suggested that discontinuation of bevacizumab might increase VEGF, resulting in more aggressive disease^18^. Several in vitro studies have suggested that, if anti-VEGF therapy fails, anti-VEGF therapy may cause more severe hypoxia in the tumor microenvironment, leading to tumor progression and greater malignancy or inducing metastasis^22–25^. While predictive factors for bevacizumab therapy remain unclear, our previous study showed that a high absolute lymphocyte count (ALC) and low neutrophil-to-lymphocyte ratio (NLR) as systemic immunity markers were independent predictive markers of PB therapy for both better TTF and OS^26^. Further prospective studies are warranted to confirm clinically useful biomarkers for selecting patients expected to benefit from PB therapy.

Eribulin, a tubulin dynamics inhibitor, improved OS in patients with HER2-ABC without prolongation of PFS compared with controls^27–30^. The discrepancy between OS and PFS has been thought to be associated with the multiple modes of actions of eribulin, such as inhibition of EMT^31^, changes in the tumor microenvironment^32,33^, and vascular remodeling^32,34^. This study demonstrated that eribulin as subsequent treatment improved OS and was an independent factor for improved OS in the shorter TTF group (HR 0.56; *P* = 0.003). In addition, in the long TTF group, subsequent eribulin administration improved OS in patients who received non-PB therapy (Wilcoxon *P* = 0.042). A biomarker for eribulin had not yet been identified in phase 3 clinical trial, the EMBRACE study^27^; however, a post-hoc analysis of the EMBRACE study and a retrospective analysis of real-world experience suggested that systemic immunity markers such as maintained peripheral lymphocytes could predict the outcomes of patients who underwent eribulin therapy^35,36^. Our results suggest that subsequent eribulin treatment might be an effective option to improve outcomes among poor responders to chemotherapy including bevacizumab or among good responders to bevacizumab if they have high absolute lymphocytes.

Our study has several limitations. First, the possibility of selection bias cannot be fully ruled out because the study was retrospectively designed. However, a strength of our study was its use of real-world data based on actual clinical practice, allowing us to compare PB therapy to conventional chemotherapy, which has not previously been compared in clinical trials. As a second limitation, the optimal cut-off values to determine “shorter TTF” or “longer TTF” remain unclear. We defined a short TTF based on the overall median TTF (183 days) of 1LCT regimens, and we referred to the results of a previous study (6 months)^10^. Third, the predictive markers for patients who do not respond to first-line PB therapy are as yet unclear. As previously mentioned, we demonstrated that high ALC and low NLR at the initiation of PB therapy were associated with better outcomes; however, we could not evaluate this relationship due to limited data availability^26^. Finally, the main limitation of this study is that we analyzed both ER-positive and ER-negative populations together. However, a retrospective study reported no significant difference in the median OS after initiation of chemotherapy between endocrine therapy-first and chemotherapy-first groups of ER-positive HER2-ABC patients. Furthermore, patients with recurrent ER-negative HER2- and patients with recurrent ER-positive HER2- who underwent sequential chemotherapy had an almost identical OS after initiation of chemotherapy^37^. Therefore, to minimize the influence of ER-expression on OS, we defined OS not as the time from diagnosis of ABC but instead from the initiation of 1LCT to the date of death from any cause. We then performed the multivariate analyses including the factors of ER and endocrine therapy after 1LCT. Notably, inhibitors of cyclin-dependent kinases 4 and 6 combined with endocrine therapy are the standard therapy for ER-positive HER2-ABC^7^. Since inhibitors of cyclin-dependent kinases 4 and 6 may prolong OS in ER-positive ABC patients^6^, we believe that this study is a valuable contribution in the era without inhibitors of cyclin-dependent kinases 4 and 6.

In this study, we investigated patients with HER2-ABC with a focus on sensitivity to 1LCT. We found that a shorter duration of 1LCT, particularly in PB therapy, was associated with inferior OS; however, subsequent eribulin therapy significantly improved OS in patients who were poor responders to 1LCT including PB therapy. We still need confirmed biomarkers that predict the effect of a treatment such as ALC but also that help us reconsider the strategy in the middle of treatment, and these biomarkers will lead to improvements in OS. Further research is needed to confirm these biomarkers.

## Methods

### Patients

We used the same dataset as in our previous study and the details of the study have been published^11^. Briefly, we retrospectively analyzed 301 patients with HER2-ABC who received the 1LCT at Fukuyama City Hospital, Hiroshima City Hiroshima Citizens Hospital, and Shizuoka Cancer Center between January 2011 and December 2016. Tumor assessment was performed according to the Response Evaluation Criteria in Solid Tumors (version 1.1)^38^ at intervals based on clinical judgment.

This retrospective study was approved by the institutional review boards of each participating institution (approval number: Fukuyama City Hospital: 359; Hiroshima City Hiroshima Citizens Hospital: 2019-73; and Shizuoka Cancer Center: T30-25). All procedures that involved human participants were conducted in accordance with the ethical standards of the institutional and/or national research committees and compliance with the 1964 Declaration of Helsinki and its later amendments or other comparable ethical standards. Informed consent was obtained in the form of an opt-out option on the hospital website from all individual participants of this study.

### Treatments

First-line and subsequent chemotherapy regimens, dose modifications, and interruptions or discontinuations were determined based on the physician’s judgment and/or patient preferences as a routine practice in clinical studies^11^. In our study, TTF was defined as the time from the administration of 1LCT to the discontinuation of treatment for any reason, including disease progression, treatment toxicity, patient/physician decision, or death from any cause. The patients were divided into two groups: longer TTF and shorter TTF, according to the TTF of 1LCT in each patient. The cut-off value for TTF was set at 183 days, which was based on the overall median TTF of 1LCT in this study. These two groups were subclassified into two additional groups based on the 1LCT regimen received: PB and non-PB regimens. The non-PB group included anthracycline (such as epirubicin/cyclophosphamide), taxane (such as docetaxel), eribulin, 5-fluorouracil (such as capecitabine, S-1 [combination drug of Tegafur, Gimeracil, and Oteracil Potassium]), and “other” (such as vinorelbine and gemcitabine)^11^.

We used the same definition as in our previous study^11^. OS was defined as the time from the administration of 1LCT to the date of death from any cause. The ORR was defined as the percent of patients who achieved a complete response or partial response. The clinical benefit rate was defined as the percent of patients who achieved a complete response or partial response or maintained stable disease for > 24 weeks.

### Statistical analyses

We used Wilcoxon’s rank-sum test to compare the median age and Fisher’s exact tests to compare the proportions of categorical variables. Survival analyses were estimated using the Kaplan–Meier method. Comparisons between the groups were performed using a log-rank test or generalized Wilcoxon test. We used Cox regression models for the univariate and multivariate analyses. Logistic regression was performed to identify risk factors for short TTF of 1LCT. We included the baseline patient characteristics with P < 0.10 in the univariate analysis in the multivariate analysis. Spearman’s rank correlation coefficient (ρ ↑　(Spearman's ρ )) was employed to study the association between TTF and survival. In all statistical analyses, P < 0.05 was considered to be statistically significant. These analyses were performed using the EZR software program (Saitama Medical Center, Jichi Medical University, Saitama, Japan), which is a graphical user interface for the R software program (The R Foundation for Statistical Computing, Vienna, Austria)^39^.

## Supplementary Information

Supplementary Information.

## Data Availability

The data sets obtained and/or analyzed during the current study can be made available by the corresponding author on a reasonable request.

## References

[1] Partridge AH (2014). Chemotherapy and targeted therapy for women with human epidermal growth factor receptor 2-negative (or unknown) advanced breast cancer: American Society of Clinical Oncology Clinical Practice Guideline. J. Clin. Oncol..

[2] Broglio KR, Berry DA (2009). Detecting an overall survival benefit that is derived from progression-free survival. J. Natl. Cancer Inst..

[3] Burzykowski T (2008). Evaluation of tumor response, disease control, progression-free survival, and time to progression as potential surrogate end points in metastatic breast cancer. J. Clin. Oncol..

[4] Adunlin G, Cyrus JW, Dranitsaris G (2015). Correlation between progression-free survival and overall survival in metastatic breast cancer patients receiving anthracyclines, taxanes, or targeted therapies: a trial-level meta-analysis. Breast Cancer Res. Treat..

[5] Swain SM (2015). Pertuzumab, trastuzumab, and docetaxel in HER2-positive metastatic breast cancer. N. Engl. J. Med..

[6] DeMichele A (2021). Comparative effectiveness of first-line palbociclib plus letrozole versus letrozole alone for HR+/HER2- metastatic breast cancer in US real-world clinical practice. Breast Cancer Res..

[7] Cardoso F (2020). 5th ESO-ESMO international consensus guidelines for advanced breast cancer (ABC 5). Ann. Oncol..

[8] Delaloge S (2016). Paclitaxel plus bevacizumab or paclitaxel as first-line treatment for HER2-negative metastatic breast cancer in a multicenter national observational study. Ann. Oncol..

[9] Gennari A (2011). Duration of chemotherapy for metastatic breast cancer: a systematic review and meta-analysis of randomized clinical trials. J. Clin. Oncol..

[10] Yamamura J (2019). Response to first-line recurrence treatment influences survival in hormone receptor-positive, HER2-negative breast cancer: A multicenter study. In Vivo.

[11] Nakamoto S, Watanabe J, Ohtani S, Morita S, Ikeda M (2020). Bevacizumab as first-line treatment for HER2-negative advanced breast cancer: Paclitaxel plus bevacizumab versus other chemotherapy. In Vivo.

[12] Sherrill B (2008). Relationship between effects on time-to-disease progression and overall survival in studies of metastatic breast cancer. Br. J. Cancer.

[13] Tang PA, Bentzen SM, Chen EX, Siu LL (2007). Surrogate end points for median overall survival in metastatic colorectal cancer: literature-based analysis from 39 randomized controlled trials of first-line chemotherapy. J. Clin. Oncol..

[14] Buyse M (2007). Progression-free survival is a surrogate for survival in advanced colorectal cancer. J. Clin. Oncol..

[15] Hackshaw A, Knight A, Barrett-Lee P, Leonard R (2005). Surrogate markers and survival in women receiving first-line combination anthracycline chemotherapy for advanced breast cancer. Br. J. Cancer.

[16] Vasan N, Baselga J, Hyman DM (2019). A view on drug resistance in cancer. Nature.

[17] Lainetti PF, Leis-Filho AF, Laufer-Amorim R, Battazza A, Fonseca-Alves CE (2020). Mechanisms of resistance to chemotherapy in breast cancer and possible targets in drug delivery systems. Pharmaceutics.

[18] Miller K (2007). Paclitaxel plus bevacizumab versus paclitaxel alone for metastatic breast cancer. N. Engl. J. Med..

[19] Ciruelos E, Pérez-García JM, Gavilá J, Rodríguez A, de la Haba-Rodriguez J (2019). Maintenance therapy in HER2-negative metastatic breast cancer: A new approach for an old concept. Clin. Drug Investig..

[20] Zielinski C (2016). Bevacizumab plus paclitaxel versus bevacizumab plus capecitabine as first-line treatment for HER2-negative metastatic breast cancer (TURANDOT): primary endpoint results of a randomised, open-label, non-inferiority, phase 3 trial. Lancet Oncol..

[21] Miles D (2017). Bevacizumab plus paclitaxel versus placebo plus paclitaxel as first-line therapy for HER2-negative metastatic breast cancer (MERiDiAN): A double-blind placebo-controlled randomised phase III trial with prospective biomarker evaluation. Eur. J. Cancer.

[22] Ueda S (2014). Optical imaging for monitoring tumor oxygenation response after initiation of single-agent bevacizumab followed by cytotoxic chemotherapy in breast cancer patients. PLoS ONE.

[23] Ebos JM (2009). Accelerated metastasis after short-term treatment with a potent inhibitor of tumor angiogenesis. Cancer Cell.

[24] Pàez-Ribes M (2009). Antiangiogenic therapy elicits malignant progression of tumors to increased local invasion and distant metastasis. Cancer Cell.

[25] Goel S (2011). Normalization of the vasculature for treatment of cancer and other diseases. Physiol. Rev..

[26] Nakamoto S (2021). Systemic immunity markers associated with lymphocytes predict the survival benefit from paclitaxel plus bevacizumab in HER2 negative advanced breast cancer. Sci. Rep..

[27] Cortes J (2011). Eribulin monotherapy versus treatment of physician's choice in patients with metastatic breast cancer (EMBRACE): a phase 3 open-label randomised study. Lancet.

[28] Kaufman PA (2015). Phase III open-label randomized study of eribulin mesylate versus capecitabine in patients with locally advanced or metastatic breast cancer previously treated with an anthracycline and a taxane. J. Clin. Oncol..

[29] Twelves C (2014). Efficacy of eribulin in women with metastatic breast cancer: A pooled analysis of two phase 3 studies. Breast Cancer Res. Treat..

[30] Pivot X (2016). Pooled analyses of eribulin in metastatic breast cancer patients with at least one prior chemotherapy. Ann. Oncol..

[31] Yoshida T (2014). Eribulin mesilate suppresses experimental metastasis of breast cancer cells by reversing phenotype from epithelial-mesenchymal transition (EMT) to mesenchymal-epithelial transition (MET) states. Br. J. Cancer.

[32] Funahashi Y (2014). Eribulin mesylate reduces tumor microenvironment abnormality by vascular remodeling in preclinical human breast cancer models. Cancer Sci..

[33] Ito K (2017). Antitumor effects of eribulin depend on modulation of the tumor microenvironment by vascular remodeling in mouse models. Cancer Sci..

[34] Ueda S (2016). In vivo imaging of eribulin-induced reoxygenation in advanced breast cancer patients: a comparison to bevacizumab. Br. J. Cancer.

[35] Watanabe J, Saito M, Horimoto Y, Nakamoto S (2020). A maintained absolute lymphocyte count predicts the overall survival benefit from eribulin therapy, including eribulin re-administration, in HER2-negative advanced breast cancer patients: A single-institutional experience. Breast Cancer Res. Treat..

[36] Miyoshi Y (2020). High absolute lymphocyte counts are associated with longer overall survival in patients with metastatic breast cancer treated with eribulin-but not with treatment of physician's choice-in the EMBRACE study. Breast Cancer.

[37] Watanabe J, Hayashi T, Tadokoro Y, Nishimura S, Takahashi K (2017). Clinical pattern of primary systemic therapy and outcomes of estrogen receptor-positive, HER2-negative metastatic breast cancer: A review of a single institution. Breast Cancer Res. Treat..

[38] Eisenhauer EA (2009). New response evaluation criteria in solid tumours: revised RECIST guideline (version 1.1). Eur. J. Cancer.

[39] Kanda Y (2013). Investigation of the freely available easy-to-use software 'EZR' for medical statistics. Bone Marrow Transplant..

