# Supplementary material for: Shorter duration of first-line chemotherapy reflects poorer outcomes in patients with HER2-negative advanced breast cancer: a multicenter retrospective study
Source: Sci Rep. 2021 Nov 2;11:21454. doi: 10.1038/s41598-021-00711-x (PMC8563944; doi:10.1038/s41598-021-00711-x)
Supplement: Supplementary file 1 — Supplementary Information. [file 41598_2021_711_MOESM1_ESM.docx]

**Shorter duration of first-line chemotherapy reflects poorer outcomes in patients with HER2-negative advanced breast cancer: A multicenter retrospective study**

Shogo Nakamoto^1,2,*^, Junichiro Watanabe^1,3^, Shoichiro Ohtani^4^, Satoshi Morita^5^, Masahiko Ikeda^2^

^1^　Division of Breast Oncology, Shizuoka Cancer Center, 1007 Shimonagakubo, Nagaizumi, Shizuoka 411-8777, Japan

^2^ Division of Breast and Thyroid Gland Surgery, Fukuyama City Hospital, 5-23-1 Zao, Fukuyama, Hiroshima 721-8511, Japan

^3^ Department of Breast Oncology, Juntendo University School of Medicine, 3‑1‑3 Hongo, Bunkyo‑ku, Tokyo 113‑8431, Japan

^4^　Division of Breast Surgery, Hiroshima City Hiroshima Citizens Hospital, 7-33 Motomachi, Naka-ku, Hiroshima, Hiroshima730-8518, Japan

^5^ Division of Biomedical Statistics and Bioinformatics, Kyoto University Graduate School of Medicine, Yoshida-Konoe, Sakyo-ku, Kyoto 606-8501, Japan

**Corresponding author:** Shogo Nakamoto

Division of Breast and Thyroid Gland Surgery, Fukuyama City Hospital

5-23-1 Zao, Fukuyama, Hiroshima, Japan, 7218511

E-mail address: p92c9f20@s.okayama-u.ac.jp

**Supplementary Table S1.** Patient characteristics at the baseline by first-line chemotherapy regimen

| Characteristics, n (%) | PB group (n = 114) | | *P* value | non-PB group (n = 187) | | *P* value |
| --- | --- | --- | --- | --- | --- | --- |
|  | short TTF (n = 46) | long TTF (n = 68) |  | short TTF (n = 103) | long TTF (n = 84) |  |
| Median age, years (range) | 53 (29–76) | 59 (29–83) | 0.027^a^ | 60 (28–87) | 61 (37–90) | 0.19^a^ |
| ≥60 years | 15 (32.6) | 32 (47.1) | 0.17 | 54 (52.4) | 45 (53.6) | 0.88 |
| Estrogen receptor status |  |  |  |  |  |  |
| Positive | 27 (58.7) | 50 (73.5) | 0.051^b^ | 72 (70.6) | 62 (73.7) | 0.24^b^ |
| Negative | 17 (37.0) | 13 (19.1) |  | 30 (31.5) | 18 (20.4) |  |
| Unknown | 2 (4.3) | 5 (7.4) |  | 1 (2.0) | 4 (5.9) |  |
| Diagnosis |  |  |  |  |  |  |
| *De novo* | 21 (45.7) | 26 (38.2) | 0.45 | 30 (29.1) | 18 (21.4) | 0.24 |
| Recurrence | 25 (54.3) | 42 (61.8) |  | 73 (70.9) | 66 (78.6) |  |
| Metastases |  |  |  |  |  |  |
| Central nervous system | 4 (8.7) | 3 (4.4) | 0.44 | 4 (3.9) | 6 (7.1) | 0.35 |
| Bone | 29 (63.0) | 39 (57.4) | 0.57 | 60 (58.3) | 48 (57.1) | 0.88 |
| Lung | 14 (30.4) | 30 (44.1) | 0.17 | 37 (35.9) | 31 (36.9) | 1.00 |
| Pleura/ lymphangiopathy | 10 (21.7) | 21 (30.9) | 0.39 | 23 (22.3) | 13 (15.5) | 0.27 |
| Lymph node | 37 (80.4) | 52 (76.5) | 0.65 | 68 (66.0) | 49 (58.3) | 0.29 |
| Liver | 24 (52.2) | 31 (45.6) | 0.57 | 29 (28.2) | 26 (31.0) | 0.75 |
| Type of metastases |  |  |  |  |  |  |
| Visceral | 32 (69.6) | 48 (70.6) | 1.00 | 63 (61.2) | 51 (60.7) | 1.00 |
| Non-visceral | 14 (30.4) | 20 (29.4) |  | 40 (38.8) | 33 (39.3) |  |
| Number of metastatic sites | |  |  |  |  |  |
| ≥3 | 30 (65.2) | 46 (67.6) | 0.84 | 61 (59.2) | 38 (45.2) | 0.077 |
| <3 | 16 (34.8) | 22 (32.4) |  | 42 (40.8) | 46 (54.8) |  |
| Perioperative chemotherapies ^c^ | |  |  |  |  |  |
| Yes | 21 (45.7) | 27 (39.7) | 0.57 | 54 (52.4) | 45 (53.6) | 0.88 |
| No | 25 (54.3) | 41 (60.3) |  | 49 (47.6) | 39 (46.4) |  |
| Disease-free interval |  |  |  |  |  |  |
| <24 months | 36 (78.3) | 38 (55.9) | 0.017 | 64 (62.1) | 36 (42.9) | 0.012 |
| ≥24 months | 10 (21.7) | 30 (44.1) |  | 39 (37.9) | 48 (72.7) |  |
| Prior endocrine therapy ^d^ | 6 (22.2) | 20 (40.0) | 0.044 | 45 (62.5) | 48 (77.4) | 0.078 |
| Response of first-line therapy | |  |  |  |  |  |
| Overall response rate | 26 (56.5) | 60 (88.2) | <0.001 | 23 (22.3) | 41 (48.8) | <0.001 |
| Clinical benefit rate | 26 (56.5) | 67 (98.5) | <0.001 | 25 (24.3) | 81 (96.4) | <0.001 |

a. Wilcoxon’s rank sum test was performed

b. Comparison of estrogen receptor positive and estrogen receptor negative

c Treatment with anthracycline and/or taxane

d. Endocrine therapy for advanced breast cancer treatment before first-line chemotherapy in patients with estrogen receptor positive

Abbreviations: PB, paclitaxel plus bevacizumab; TTF, time to treatment failure

**Supplementary Table S2.** The relative risk of longer time to treatment failure of first-line chemotherapy according to patient and tumor characteristics’ regimens (logistic regression)

| Characteristics | PB group | | | non-PB group | | |
| --- | --- | --- | --- | --- | --- | --- |
|  | OR | 95% CI | P value | OR | 95% CI | P value |
| Age at first-line chemotherapy | 1.01 | 0.97–1.05 | 0.15 | - | - | - |
| Estrogen receptor negative | 0.67 | 0.24–1.88 | 0.45 | 0.97 | 0.42–2.24 | 0.94 |
| ≥3 metastatic sites | - | - | - | 0.47 | 0.24–0.90 | 0.023 |
| Disease-free interval <24 months | 0.27 | 0.08–0.84 | 0.024 | 0.58 | 0.30–1.12 | 0.10 |
| Prior endocrine therapy ^a^ | 3.21 | 0.87–11.9 | 0.080 | 1.77 | 0.83–3.79 | 0.14 |
| ORR of first-line therapy | 12.5 | 3.57–44.0 | <0.001 | 3.94 | 1.96–7.91 | <0.001 |

a. Endocrine therapy for advanced breast cancer treatment before first-line chemotherapy

Abbreviations: CI, confidence interval; OR, Odd ratio; ORR, overall response rate; PB, paclitaxel and bevacizumab**Supplementary Table S3.** Univariate and multivariate analyses of the overall survival in patients in the longer time to treatment failure group (Cox hazard model)

| Variables | Univariate | | | Multivariate | | |
| --- | --- | --- | --- | --- | --- | --- |
|  | HR | 95% CI | *P* | HR | 95% CI | *P* |
| Age (≥60 vs. <60 years) | 1.56 | 1.04–2.33 | 0.031 | 1.42 | 0.95–2.13 | 0.088 |
| Estrogen receptor (negative vs. positive) | 1.05 | 0.64–1.72 | 0.85 | - | - | - |
| Diagnosis (recurrence vs. advanced) | 0.97 | 0.62–1.52 | 0.89 | - | - | - |
| Metastatic sites (yes vs. no) |  |  |  |  |  |  |
| Central nervous system | 0.48 | 0.15–1.53 | 0.22 | - | - | - |
| Bone | 1.36 | 0.90–2.05 | 0.14 | - | - | - |
| Lungs | 1.04 | 0.69–1.56 | 0.85 | - | - | - |
| Pleura and/or lymphangiopathy | 1.08 | 0.66–1.78 | 0.75 | - | - | - |
| Lymph nodes | 1.00 | 0.65–1.53 | 1.00 | - | - | - |
| Liver | 1.87 | 1.23–2.83 | 0.003 | 1.88 | 1.24–2.85 | 0.003 |
| Visceral metastasis (yes vs. no) | 1.39 | 0.90–2.15 | 0.14 | - | - | - |
| Number of metastatic sites (≥3 vs. <3) | 1.27 | 0.85–1.90 | 0.25 | - | - | - |
| Perioperative chemotherapy ^a^ (yes vs. no) | 1.00 | 0.67–1.50 | 1.00 | - | - | - |
| Disease-free interval (<24 months vs. ≥24) | 1.03 | 0.69–1.53 | 0.90 | - | - | - |
| First-line chemotherapy regimens (PB vs. non-PB) | 0.80 | 0.52–1.22 | 0.29 | - | - | - |
| Eribulin as subsequent therapy (yes vs. no) | 1.09 | 0.73–1.65 | 0.67 | - | - | - |
| Endocrine therapy after first-line chemotherapy (yes vs. no) | 0.54 | 0.36–0.81 | 0.003 | 0.55 | 0.36–0.83 | 0.004 |

Abbreviations: CI, confidence interval; HR, hazard ratio; PB, paclitaxel and bevacizumab
